# First symptoms in multiple system atrophy

**DOI:** 10.1007/s10286-017-0500-0

**Published:** 2018-01-08

**Authors:** Jake H. McKay, William P. Cheshire

**Affiliations:** Department of Neurology, Mayo Clinic, 4500 San Pablo Rd., Jacksonville, FL 32224 USA

**Keywords:** Multiple system atrophy, Autonomic nervous system diseases, Symptom assessment, Early diagnosis

## Abstract

**Purpose:**

The initial symptoms of multiple system atrophy (MSA) and, in particular, early autonomic symptoms, have received less attention than motor symptoms. Whereas pathognomonic motor signs are essential to diagnostic specificity, early symptoms important to recognition of a neurodegenerative disorder may be less apparent or diagnostically ambiguous. This observational study sought to identify the very earliest symptoms in the natural history of MSA.

**Methods:**

Detailed clinical histories focusing on early symptoms were obtained from 30 subjects recently diagnosed with MSA. Historical data were correlated with neurological examinations and laboratory autonomic testing.

**Results:**

Subjects’ mean age was 63.9 years. Ten were classified as having MSA-P and 20 MSA-C. The evaluations occurred 2.9 ± 0.4 months after diagnosis. The first symptom of MSA was autonomic in 22 (73%) and motor in 3 (10%) subjects (*p* < 0.0001). The most frequent first symptom was erectile failure, which occurred in all men beginning 4.2 ± 2.6 years prior to diagnosis. After erectile failure, postural lightheadness or fatigue following exercise, urinary urgency or hesitancy, and violent dream enactment behavior consistent with REM behavioral sleep disorder were the most frequent initial symptoms. Neither the order of symptom progression, which was highly variable, nor autonomic severity scores differentiated between MSA-P and MSA-C.

**Conclusions:**

The first symptoms of MSA are frequently autonomic and may predate recognition of motor manifestations. Orthostatic hypotension and, in men, erectile failure are among the first symptoms that, when evaluated in the context of associated clinical findings, may facilitate accurate and earlier diagnosis.

## Introduction

Multiple system atrophy (MSA) is a rare, sporadic, and ultimately fatal α-synucleinopathy with features of parkinsonism or cerebellar dysfunction, autonomic failure, and pyramidal dysfunction occurring in various combinations [1, 2]. Epidemiologic studies have found the prevalence of MSA to range from 1.9 to 3.4 per 100,000 people [3–5]. MSA affects men and women equally, with an average age of onset of approximately 55 years [2, 3]. The mean life expectancy following diagnosis is 7 years [6].

The diagnosis of MSA is often delayed [7] because its presenting signs and symptoms emerge gradually and may closely resemble those of more common disorders such as Parkinson’s disease (PD). Additionally, it is not unusual for key autonomic symptoms to be overlooked or not recognized as being potentially serious. MSA is diagnosed by clinical criteria that rely on neurological signs and symptoms. As MSA is categorized according to its extrapyramidal motor deficits into MSA-cerebellar (MSA-C) and MSA-parkinsonian (MSA-P) subtypes, its nonmotor and, in particular, autonomic features have received relatively less attention. Autonomic features, such as orthostatic hypotension (OH), urinary bladder dysfunction, and sexual dysfunction, are important aspects of MSA, both diagnostically, as specified in current consensus criteria [1], and symptomatically, as they impact patients’ quality of life.

Clinical attention typically focuses on signs and symptoms that are present and have become well-established at the time of evaluation. Early symptoms that, in retrospect, predate the clinical evaluation may not be explored in detail, yet could be important toward understanding the natural history of the disease. Whereas symptom scales that have been developed for use in MSA clinical trials are designed to monitor the progression of existing symptoms going forward in time [8], these questionnaires lack discriminative value for identifying and assessing very early symptoms that precede the diagnosis.

We sought to fill the knowledge gap regarding very early initial symptoms by performing detailed clinical histories in a cohort of recently diagnosed MSA patients. Rather than focusing only on present symptoms, we thoroughly probed for symptoms that may have initially gone unrecognized as neurologically relevant, but which potentially could supply important clues to the earliest clinical manifestations of this disorder. To that end, we applied what is considered to be the most useful of autonomic tests, a careful and precise medical history [9]. We also ascertained the temporal sequence of symptom onset and correlated symptom histories with laboratory autonomic testing.

## Methods

### Subjects

Potential subjects were identified from among 42 patients referred to the neurology clinic between July 2011 and August 2012 for consideration for participation in a randomized controlled trial of rifampicin [10], which, by design, sought to enroll patients early in the course of their disease. Subjects meeting the clinical consensus criteria [1] for a diagnosis of MSA were included.

### Clinical histories

As part of the screening process, detailed clinical histories were obtained from subjects and supplemented with input from their family members. Subjects were queried about the time of onset before diagnosis of MSA regarding symptoms of dysarthria, tremor, slow gait, loss of hand coordination, postural instability, dysphagia, dysphonia, sleep-related respiratory symptoms, dream enactment behavior, paresthesia, disturbance in the sense of smell, transient loss of consciousness, urinary hesitancy, fatigue after exercise, postural lightheadedness, urinary incontinence, constipation, urinary urgency, heat intolerance, anhidrosis, and erectile failure (in men). Sleep habits were ascertained by interviewing subjects’ bedpartners or family members, who were questioned regarding snoring, apnea, and stridor. Subjects were considered to have probable REM behavioral sleep disorder if there was a history of recurrent violent behaviors or shouting during sleep. All but five, who were evaluated by a movement disorders subspecialist, were examined by the senior author of this paper (WPC). The early symptoms predated use of dopaminergic therapy.

As this retrospective review was conducted 5 years following enrollment and the gathering of historical data, medical records were reviewed again for any interval changes in diagnosis.

### Autonomic testing

All subjects underwent neurological examinations as well as autonomic testing consisting of a battery of standardized tests performed under controlled conditions. Subjects were instructed not to consume food, caffeine, or nicotine for 3 h prior to testing. Anticholinergic and cardiovascular medications were discontinued for five half-lives prior to testing.

Postganglionic sudomotor responses were assessed at the forearm, proximal leg, distal leg, and foot by Q-Sweat^®^ (WR Medical, Maplewood, MN, USA). Continuous blood pressure (BP) and heart rate (HR) monitoring was conducted noninvasively using Nexfin^®^ optical plethysmometry (Bmeye, Amsterdam, The Netherlands) in the left index or middle finger. BP was recorded simultaneously in the right arm by intermittent sphygmomanometry. Heart rate variability to deep breathing was assessed in the supine position at a respiratory frequency of 6 Hz under continuous electrocardiography registration. While in the recumbent position, subjects performed a series of Valsalva maneuvers by maintaining a steady expiratory force of 40 mm Hg for 15 s while blowing into a plastic bugle punctured to create a tiny air leak. The Valsalva ratio was defined as the maximum heart rate during phase II divided by the minimum heart rate during phase IV. Adrenergic function was assessed by measuring the recovery of BP during late phase II, the pressure recovery time during phase IV, and the overshoot of BP during phase IV. Subjects then underwent passive upright tilt table testing to 70°. OH was defined as a sustained reduction of systolic BP of at least 20 mm Hg or diastolic BP of 10 mm Hg within 3 min of head-up tilt [11].

Autonomic test results for sudomotor, cardiovagal, and adrenergic function were scored and combined into the Composite Autonomic Severity Score (CASS) [12]. Autonomic system subscores as well as orthostatic BP measurements and Valsalva pressure recovery times were correlated with clinical histories using analysis of variance with statistical significance defined as *p* < 0.05.

## Results

### Patient characteristics

Of the 42 subjects who underwent screening, the diagnostic criteria for MSA were met in 35, of whom 9 were classified as possible and 26 probable by consensus criteria [1]. Twelve did not meet the diagnostic criteria for MSA and were excluded, being diagnosed instead with dementia with Lewy bodies (DLB), PD, or spinocerebellar ataxia.

At the time of retrospective review 5 years after enrollment, the clinical diagnosis of MSA was retained or had become more certain for a majority, but in 5 had been revised from MSA (possible in 1 and probable in 4) to DLB based on follow-up examinations, cognitive decline, and visual hallucinations. The remaining subjects were classified as 8 possible, 18 probable, and 4 definite by pathological confirmation. The revised number of subjects with MSA was 30. Of these, 10 were classified as MSA-P and 20 MSA-C. Their median age was 63.9 years (range 39–86), and 8 (27%) were female. The time at evaluation (when detailed histories were obtained) was 2.9 ± 0.4 (mean ± SD) months (range 0–15 months) following initial diagnosis. Eighteen subjects received the first diagnosis of MSA at the time of evaluation.

### Symptoms

**Fig. 1:**
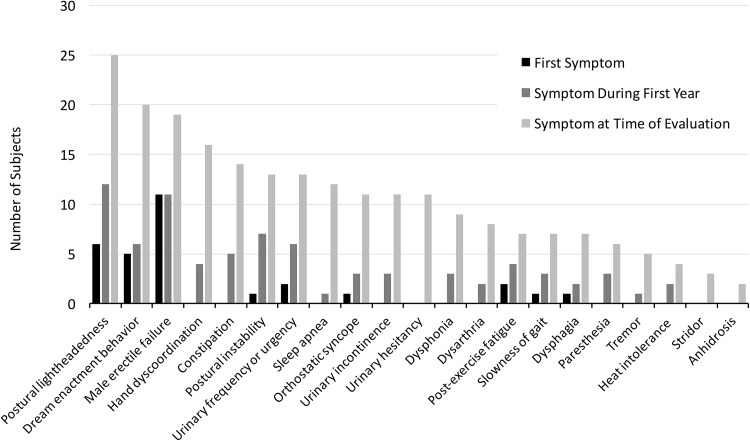
Number of MSA subjects from the cohort of 30 subjects reporting specific symptoms: as the very first symptom (black), during the first symptomatic year (medium gray), and at the time of evaluation (light gray)

The frequencies of specific symptoms that appeared initially, that were present during the first symptomatic year, and that were present at the time of evaluation are shown alongside one another in Fig. 1. The first presenting symptom of MSA that was recalled in retrospect was autonomic in 22 (73%), sleep-related in 5 (17%), and motor in 3 (10%) of subjects. Excluding sleep-related initial symptoms, the first symptom was autonomic in 25, motor in 4, and concomitantly autonomic and motor in 1 (*p* < 0.0001). Subjects whose first symptoms were autonomic had noted them 5.5 ± 3.7 years earlier, as compared to 4.0 ± 1.7 years in subjects whose first symptoms were motor nonsignificant. Duration of symptoms at the time of diagnosis is presented in Table 1.

**Table 1:** MSA symptoms that were present at the time of evaluation and their duration prior to diagnosis

Category	Symptom	Duration (mean years ± SD)
Autonomic	Urinary frequency or urgency	4.1 ± 4.7
	Heat intolerance	4.1 ± 4.7
	Male erectile failure	4.0 ± 2.6
	Post-exercise fatigue	3.0 ± 2.9
	Postural lightheadedness	2.8 ± 3.2
	Orthostatic syncope	2.0 ± 1.6
	Urinary hesitancy	2.0 ± 1.6
	Urinary incontinence	1.7 ± 0.8
	Constipation	1.6 ± 1.4
	Anhidrosis	0.6 ± 0.1
Motor	Slowness of gait	3.2 ± 2.1
	Postural instability	2.7 ± 2.5
	Tremor	2.6 ± 2.6
	Hand dyscoordination	2.0 ± 2.1
	Dysphonia	1.2 ± 1.2
	Dysphagia	1.1 ± 1.0
	Dysarthria	0.8 ± 0.6
	Dream enactment behavior	3.2 ± 2.7
Sleep	Sleep apnea	1.4 ± 1.1
	Stridor	1.0 ± 0.1
Sensory	Paresthesia	1.7 ± 1.2

The most frequent initial symptom was complete erectile failure, which was the first symptom in 12 and was eventually present in 100% of the 19 men who were asked about this. The second most frequent initial symptom was postural lightheadedness, which was the first symptom in 6 (21%) and was eventually present in 25 (89%) of the 28 subjects who were asked. The third most frequent symptom was violent dream enactment behavior suggesting REM behavioral sleep disorder, occurring in 5 (19%), confirmed by polysomnography in 2, and was eventually present in 20 (77%) of those asked. The fourth most frequent symptom was post-exercise fatigue, occurring in 3 (17%), and was eventually present in 7 (39%) of those asked. The fifth most frequent symptom was urinary urgency, occurring in 2 (10%), and was eventually present in 13 (62%) of those asked. Frequencies of all symptoms are shown in Fig. 1. The sequence in which symptoms occurred and progressed was variable with no stereotypical order of progression identified.

Nocturnal respiratory symptoms were reported in 17 (57%), all of whom had snoring. Of them, 12 (40%) also had witnessed or polysomnogram-documented apnea, and 3 (10%) had stridor. CASS scores were not significantly different for those with snoring or stridor as compared to those without.

### Autonomic testing

Analysis of the 30 MSA cases found the CASS scores to be 6.2 ± 2.3, consistent with moderately severe autonomic failure. CASS subscores for sudomotor, cardiovagal, and adrenergic failure were 2.1 ± 1.1, 1.9 ± 1.0, and 2.3 ± 1.0, respectively (on scales of 3, 3, and 4, for a maximum deficit of 10). CASS scores did not differ significantly between those with autonomic (6.3 ± 2.4) versus motor (5.5 ± 5.5) initial symptomatic onset or between those with MSA-P (5.9 ± 2.4) versus MSA-C (6.3 ± 2.3).

During head-up tilt, subjects exhibited a fall in systolic/diastolic BP of 48.8/26.2 ± 33.2/21.2 mm Hg with an increase in heart rate of 8.0 ± 7.0 beats/min. Valsalva pressure recovery times were prolonged 15.6 ± 11.1 s (normal < 5 s). Of those diagnosed with MSA, 21 met the strictest OH criterion of a decrease in systolic BP of 30 mmHg and diastolic BP of 15 mmHg within 3 min of standing [1], whereas 9 met the standard criterion of 20 or 10 mmHg, respectively [11]. Both OH categories had evidence of substantial adrenergic failure on autonomic testing with mean adrenergic scores of 2.7 versus 1.8, CASS scores of 6.7 versus 5.4, and pressure recovery times of 19 versus 10 s, respectively.

## Discussion

The results of this study indicate that early symptoms in MSA are frequently autonomic and predate recognition of motor manifestations. At the time of initial diagnosis, careful attention to historical details found that a majority (77%) of MSA patients recalled early autonomic symptoms that initially were not recognized as being a manifestation of a neurodegenerative disease. A history of erectile failure, in particular, in men was invariably present as early as 4 or more years prior to diagnosis and very often was the first symptom. For most subjects, the onset of autonomic symptoms was followed by the appearance of motor symptoms within a year.

This study highlights the importance of paying close attention to autonomic symptoms as potential early manifestations of MSA. Our findings are in keeping with those of Kaufmann and colleagues, who followed 100 patients with pure autonomic failure and found that 34% of them phenoconverted within 4 years to an α-synucleinopathy, 8% of them to MSA [13]. Moreover, our finding of a high incidence of erectile failure accords with the study by Beck and colleagues, who reported erectile impotence in 96% of 62 males with MSA, it being the first symptom in 37% [14]. Whereas erectile dysfunction can be a nonspecific symptom in men of advancing age, occurring in 11.5% of men 60–69 years of age [15], complete erectile failure, especially when progressing to failure of detrusor contraction and accompanied by OH, and either bradykinesia or ataxia, should arouse clinical suspicion for possible MSA. Conversely, in our opinion, the absence of erectile dysfunction in a patient with signs of parkinsonism or cerebellar ataxia would be a strong indication against a diagnosis of MSA.

Further, this study expands on the work of Colosimo and colleagues, who tabulated nonmotor symptoms in 172 patients with atypical parkinsonism, 34 of whom had MSA-P. They found that symptoms of urinary dysfunction and postural hypotension were most frequent in MSA-P (in 91 and 53%, respectively) as compared to other forms of parkinsonism. Their study did not explore sexual function in detail but grouped it in the category of “other symptoms” [16]. Other investigators have noted that sexual dysfunction in MSA tends to be underreported [17], and a single study found that women with MSA reported decreased genital sensitivity [18]. The reason for underreporting is partly cultural, as patients may feel uncomfortable discussing such a private matter. Assessing autonomic sexual dysfunction in women is especially challenging, as the endocrine effects of aging on the sexual organs make it difficult to know whether factors such as vaginal dryness and decreased engorgement are the result of autonomic denervation or postmenopausal atrophy. As all of the female subjects in our study were postmenopausal, detailed sexual histories were not obtained.

A history of anosmia was not present in any of the 30 MSA subjects. By contrast, all 5 from among the 35 initially suspected of having MSA who had symptoms of hyposmia or anosmia subsequently evolved into the phenotype of DLB. This finding is in keeping with several recent studies that have shown a high degree of discriminatory specificity of olfaction, which is preserved in MSA but frequently deficient in PD or DLB [13, 19, 20]. The patient with marked hyposmia may be unlikely to have MSA [19].

Several negative findings in our study are noteworthy. No consistent pattern emerged regarding the sequence of the development of autonomic or motor symptoms. The order in which multiple symptoms emerged was highly variable. Additionally, there were no significant differences in autonomic involvement comparing MSA-P to MSA-C or differences in CASS scores comparing those with early autonomic symptoms to those with early motor symptoms.

The clinical diagnosis of MSA, as distinguished from other types of parkinsonism or cerebellar ataxia, can be challenging early in the course of the disease, as the clinical features of these entities frequently overlap. One clue to MSA is that, at the time of diagnosis, autonomic dysfunction is typically more severe as compared to that in PD, and autonomic dysfunction preceding the development of parkinsonism could suggest MSA as a more likely diagnosis [2]. Increasingly, OH is recognized as a consistent hallmark of MSA [21], and urogenital symptoms may appear years before development of motor or cerebellar deficits [14, 22]. The diverse array of presenting motor, cerebellar, sleep, and autonomic symptoms of MSA suggests that the initial loci of α-synuclein pathology sufficient to produce symptoms vary from case to case.

Pathologically, MSA is characterized by α-synuclein-containing glial cytoplasmic inclusions (GCIs), which are associated with striatonigral and olivopontocerebellar atrophy [23–25]. Autopsies of MSA patients have demonstrated diverse patterns of extensive α-synuclein accumulation involving the putamen, caudate nucleus, external pallidum, substantia nigra, locus ceruleus, inferior olives, pontine nuclei, cerebellar Purkinje cells, and—of particular relevance to autonomic failure—intermediolateral cell columns and Onuf’s nucleus in the spinal cord [17]. GCI pathology has been found in the intermediolateral cell columns in 68% of cases and in Onuf’s nucleus in 64% of MSA cases [17]. OH correlates with intermediolateral cell column degeneration [17]. In a postmortem report of a patient with MSA-C, Wakabayashi and colleagues found CGIs predominantly involving the pontine nuclei and cerebellar white matter, and also involving the intermediolateral nuclei, but sparing the sympathetic ganglia [26]. Benarroch and colleagues compared the neuropathology of the brains of five MSA patients to controls and found that atrophy of hypothalamic arginine-vasopressin-synthesizing neurons in the suprachiasmatic nucleus also could be a contributing cause of autonomic failure in MSA patients [27].

Postmortem cases of early MSA are of particular interest in regard to correlations with early indications of clinical autonomic failure. Preclinical postmortem studies of MSA are exceedingly rare, however. Iranzo and colleagues reported a case with nocturnal stridor and OH, but without overt parkinsonian or cerebellar features, who at autopsy had evidence of α-synuclein-immunoreactive GCIs and severe neuronal loss with gliosis throughout the central nervous system [28]. Kon and colleagues reported the case of a 71-year-old man who died of choking from aspiration of food and was found to have extensive α-synuclein-immunoreactive CGIs widely distributed throughout the central nervous system [29]. In both of these cases, the pathology involved the intermediolateral cell columns of the spinal cord [28, 29].

A limitation of this study is that it was a retrospective analysis of subjective symptoms. The subjects, although cognitively intact, were chronically ill when their histories were taken, and their ability to recall precise details of their specific symptoms years earlier may have been incomplete. As this was an exploratory study, questions were system-specific but also open-ended to minimize the investigator’s bias.

These results potentially lay the groundwork for the development of structured questionnaires to screen patients for early manifestations of MSA. Recognition of initial autonomic symptoms may facilitate earlier diagnosis of this devastating α-synucleinopathy. One benefit to early diagnosis is eliminating the need for unnecessary medical testing or urological surgical interventions. There are reports of patients undergoing surgery for urinary incontinence that was later determined to be caused by MSA [30]. Ultimately, early identification of MSA patients would allow for improved targeting of symptomatic treatments of autonomic and motor symptoms as well as the opportunity to receive disease-modifying treatments to slow the progression of the disease, once such agents are identified and become available.

## Abbreviations

BP: Blood pressure
CASS: Composite Autonomic Severity Score
DLB: Dementia with Lewy bodies
GCI: Glial cytoplasmic inclusions
HR: Heart rate
MSA: Multiple system atrophy
MSA-C: Multiple system atrophy, cerebellar subtype
MSA-P: Multiple system atrophy, parkinsonian subtype
PD: Parkinson’s disease
REM: Rapid eye movement
